# Supplementary figures and images for: The changing epidemiology of hemorrhagic fever with renal syndrome in Southeastern China during 1963–2020: A retrospective analysis of surveillance data
Source: PLoS Negl Trop Dis. 2021 Aug 6;15(8):e0009673. doi: 10.1371/journal.pntd.0009673 (PMC8372920; doi:10.1371/journal.pntd.0009673)

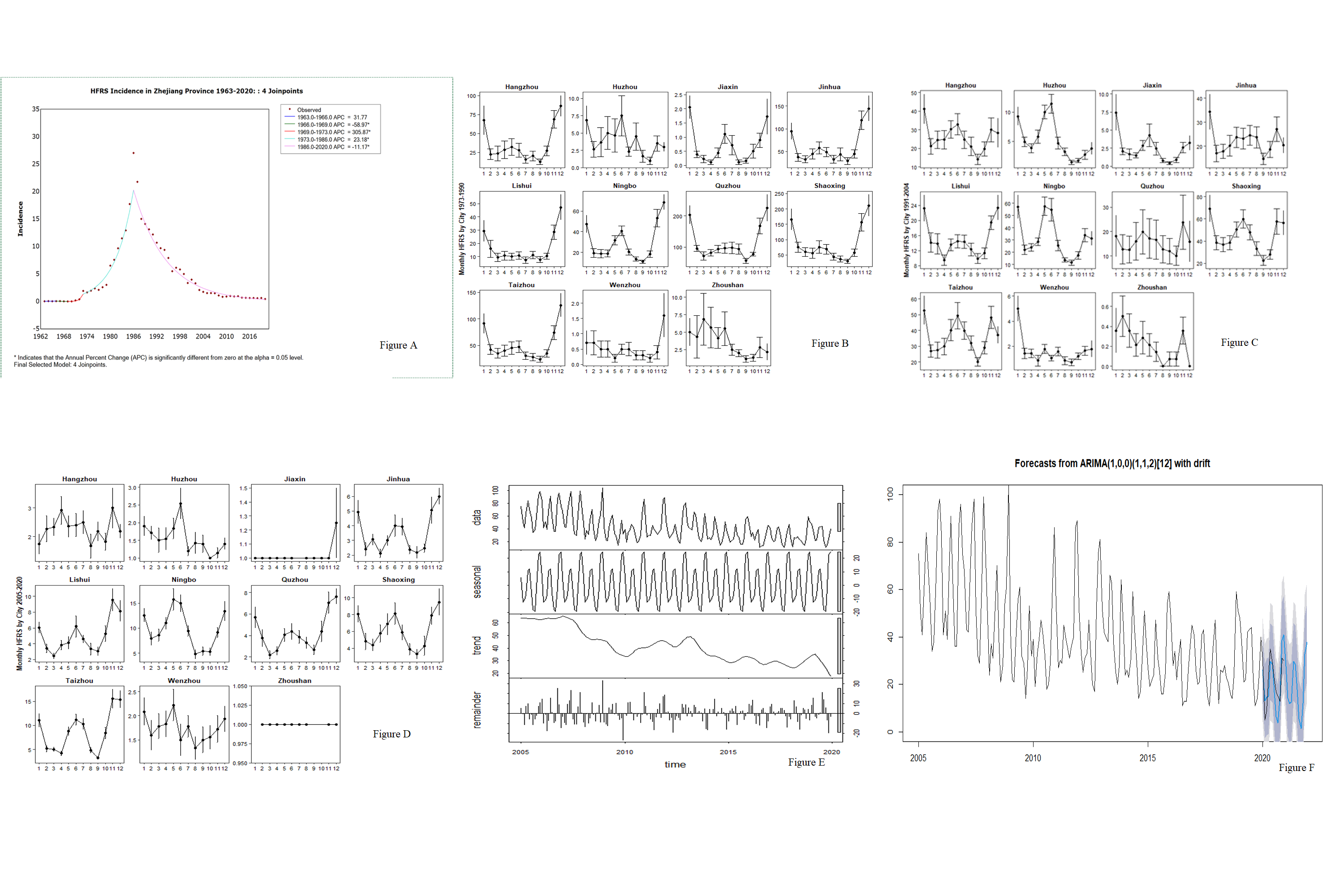

Supplement: S1 Fig — Fig A The joinpoint regression for HFRS incidence in Zhejiang Province, 1963–2020; Fig B Monthly average HFRS cases by cities in Zhejiang Province, 1973–1990; Fig C Monthly average HFRS cases by cities in Zhejiang Province, 1991–2004; Fig D Monthly average HFRS cases by cities in Zhejiang Province, 2005–2020; Fig E Seasonal decomposition for monthly HFRS in Zhejiang Province, 1963–2020; Fig F The predicted values of HFRS cases in Zhejiang Province for 2020 using SARIMA model. (TIF) [file pntd.0009673.s001.tif]
