# Supplementary material for: The changing epidemiology of hemorrhagic fever with renal syndrome in Southeastern China during 1963–2020: A retrospective analysis of surveillance data
Source: PLoS Negl Trop Dis. 2021 Aug 6;15(8):e0009673. doi: 10.1371/journal.pntd.0009673 (PMC8372920; doi:10.1371/journal.pntd.0009673)
Supplement: S1 Text — Table A Estimates of HFRS patients’ occupation in Zhejiang Province, 1991–2020; Table B Age distribution of HFRS cases in Zhejiang Province, 1991–2020. (DOC) [file pntd.0009673.s002.doc]

Table A Estimates of HFRS patients’ occupation in Zhejiang Province, 1991-2020

| **Year** | **Farmer** | **%** | **Worker** | **%** | **Housework** | **%** | **Business services** | **%** | **Students** | **%** | **Others** | **%** | **Total** |
| --- | --- | --- | --- | --- | --- | --- | --- | --- | --- | --- | --- | --- | --- |
| **1991** | 4105 | 79.19 | 409 | 7.89 | 116 | 2.24 | 45 | 0.87 | 193 | 3.72 | 316 | 6.1 | 5184 |
| **1992** | 3616 | 79.09 | 415 | 9.08 | 109 | 2.38 | 34 | 0.74 | 182 | 3.98 | 216 | 4.72 | 4572 |
| **1993** | 3290 | 78.9 | 396 | 9.5 | 62 | 1.49 | 27 | 0.65 | 158 | 3.79 | 237 | 5.68 | 4170 |
| **1994** | 3112 | 76.57 | 410 | 10.09 | 73 | 1.8 | 35 | 0.86 | 173 | 4.26 | 261 | 6.42 | 4064 |
| **1995** | 2710 | 78.53 | 302 | 8.75 | 48 | 1.39 | 25 | 0.72 | 154 | 4.46 | 212 | 6.14 | 3451 |
| **1996** | 1941 | 80.11 | 191 | 7.88 | 15 | 0.62 | 23 | 0.95 | 99 | 4.09 | 154 | 6.36 | 2423 |
| **1997** | 2224 | 81.92 | 176 | 6.48 | 29 | 1.07 | 24 | 0.88 | 107 | 3.94 | 155 | 5.71 | 2715 |
| **1998** | 2115 | 81.69 | 147 | 5.68 | 35 | 1.35 | 29 | 1.12 | 104 | 4.02 | 159 | 6.14 | 2589 |
| **1999** | 1641 | 73.59 | 292 | 13.09 | 35 | 1.57 | 22 | 0.99 | 98 | 4.39 | 142 | 6.37 | 2230 |
| **2000** | 1231 | 81.69 | 83 | 5.51 | 36 | 2.39 | 15 | 1 | 55 | 3.65 | 87 | 5.77 | 1507 |
| **2001** | 1415 | 78.92 | 119 | 6.64 | 30 | 1.67 | 15 | 0.84 | 88 | 4.91 | 126 | 7.03 | 1793 |
| **2002** | 1184 | 78.99 | 96 | 6.4 | 26 | 1.73 | 23 | 1.53 | 60 | 4 | 110 | 7.34 | 1499 |
| **2003** | 732 | 77.22 | 79 | 8.33 | 19 | 2 | 17 | 1.79 | 30 | 3.16 | 71 | 7.49 | 948 |
| **2004** | 551 | 67.28 | 135 | 16.48 | 15 | 1.83 | 9 | 1.1 | 32 | 3.91 | 77 | 9.4 | 819 |
| **2005** | 595 | 77.88 | 39 | 5.1 | 22 | 2.88 | 14 | 1.83 | 13 | 1.7 | 81 | 10.6 | 764 |
| **2006** | 597 | 78.35 | 51 | 6.69 | 23 | 3.02 | 5 | 0.66 | 21 | 2.76 | 65 | 8.53 | 762 |
| **2007** | 590 | 78.15 | 53 | 7.02 | 15 | 1.99 | 6 | 0.79 | 22 | 2.91 | 69 | 9.14 | 755 |
| **2008** | 452 | 79.3 | 36 | 6.32 | 16 | 2.81 | 6 | 1.05 | 17 | 2.98 | 43 | 7.54 | 570 |
| **2009** | 331 | 76.62 | 34 | 7.87 | 14 | 3.24 | 6 | 1.39 | 3 | 0.69 | 44 | 10.19 | 432 |
| **2010** | 333 | 71.92 | 37 | 7.99 | 14 | 3.02 | 17 | 3.67 | 12 | 2.59 | 50 | 10.8 | 463 |
| **2011** | 413 | 76.48 | 40 | 7.41 | 12 | 2.22 | 12 | 2.22 | 7 | 1.3 | 56 | 10.37 | 540 |
| **2012** | 393 | 78.6 | 34 | 6.8 | 7 | 1.4 | 9 | 1.8 | 11 | 2.2 | 46 | 9.2 | 500 |
| **2013** | 408 | 78.31 | 39 | 7.49 | 19 | 3.65 | 12 | 2.3 | 5 | 0.96 | 38 | 7.29 | 521 |
| **2014** | 289 | 75.06 | 36 | 9.35 | 12 | 3.12 | 11 | 2.86 | 7 | 1.82 | 30 | 7.79 | 385 |
| **2015** | 248 | 67.95 | 30 | 8.22 | 22 | 6.03 | 15 | 4.11 | 10 | 2.74 | 40 | 10.96 | 365 |
| **2016** | 254 | 73.41 | 22 | 6.36 | 15 | 4.34 | 9 | 2.6 | 8 | 2.31 | 38 | 10.98 | 346 |
| **2017** | 247 | 70.17 | 30 | 8.52 | 17 | 4.83 | 12 | 3.41 | 5 | 1.42 | 41 | 11.65 | 352 |
| **2018** | 237 | 70.75 | 25 | 7.46 | 22 | 6.57 | 14 | 4.18 | 5 | 1.49 | 32 | 9.55 | 335 |
| **2019** | 251 | 69.34 | 41 | 11.33 | 34 | 9.39 | 19 | 5.25 | 3 | 0.83 | 14 | 3.87 | 362 |
| **2020** | 160 | 64 | 29 | 11.6 | 32 | 12.8 | 9 | 3.6 | 10 | 4 | 10 | 4 | 250 |
| **Total** | 35665 | 78.1 | 3826 | 8.38 | 944 | 2.07 | 519 | 1.14 | 1692 | 3.71 | 3020 | 6.61 | 45666 |

Table B Age distribution of HFRS cases in Zhejiang Province, 1991-2020

|  | Cases(n=45635) | | | Deaths(n=287) | | | CFR | |
| --- | --- | --- | --- | --- | --- | --- | --- | --- |
| Age | Man | Female | Total | Man | Female | Total | Man | Female |
| <10 | 512 | 307 | 819 | 2 | 1 | 3 | 0.39 | 0.33 |
| 11-20 | 3524 | 1122 | 4646 | 11 | 5 | 16 | 0.31 | 0.45 |
| 21-30 | 7169 | 2550 | 9719 | 30 | 9 | 39 | 0.42 | 0.35 |
| 31-40 | 8533 | 3046 | 11579 | 47 | 15 | 62 | 0.55 | 0.49 |
| 41-50 | 6502 | 2618 | 9120 | 52 | 24 | 76 | 0.8 | 0.92 |
| 51-60 | 4197 | 1995 | 6192 | 34 | 21 | 55 | 0.81 | 1.05 |
| 61-70 | 1872 | 947 | 2819 | 15 | 11 | 26 | 0.8 | 1.16 |
| >70 | 486 | 255 | 741 | 8 | 2 | 10 | 1.65 | 0.78 |
| Total | 32795 | 12840 | 45635 | 199 | 88 | 287 | 0.61 | 0.69 |
