# Supplementary material for: The changing epidemiology of hemorrhagic fever with renal syndrome in Southeastern China during 1963–2020: A retrospective analysis of surveillance data
Source: PLoS Negl Trop Dis. 2021 Aug 6;15(8):e0009673. doi: 10.1371/journal.pntd.0009673 (PMC8372920; doi:10.1371/journal.pntd.0009673)
Supplement: Attachment — Submitted filename: Response.doc [file pntd.0009673.s003.doc]

Dear Editors and Reviewers,
Thank you for your comments on our manuscript entitled “The changing epidemiology of hemorrhagic fever with renal syndrome in Southeastern China during 1963-2020: a retrospective analysis of surveillance data” (PNTD-D-21-00390). Those comments are all valuable and very helpful for revising and improving our paper. We have studied comments carefully and have made correction which we hope meet with approval. All the contributing authors have reviewed the revision and concur in the revised submission.

**Respond to Reviewer #1:**
**1. Response to Methods**

1.1 What is important in this paper is massive epidemiological surveillance data on HFRS during 60 years in Zhejiang Province. Authors have to show ethical approve (approval No.) about patient’s specimens. Also, It is unclear by what criteria the clinical HFRS case was classified and lab diagnose was conducted (the ‘Diagnose criteria’ in ‘Methods’ section is not enough to explain). More detailed explanations were needed.

**Respond**: This study was reviewed and approved by the Ethics Committee of the Zhejiang Provincial Center for Disease Control and Prevention (No.2020-021). All the data of the individuals were kept confidential as requested and ethical approve.

According to the health industry standard of the People's Republic of China for diagnostic criteria of HFRS, HFRS cases were classified as suspected cases, clinically diagnosed cases and confirmed cases.

(1) A patient with epidemiological history and clinical manifestations with fever or gastrointestinal symptoms was defined as a suspected case.

(2) A suspected case with hypotension, renal function impairment, increased peripheral blood cell counts and thrombocytopenia, and a positive urine protein was defined as a clinically diagnosed case.

(3) A clinically diagnosed case with one or more of the following criteria: the serum specific IgM antibody is positive; the specific IgG antibody is 4 times higher than that in the acute phase; Hantavirus RNA is positive or Hantavirus is isolated was defined as a confirmed case.

We added these sentences in the method section.

**1.2** “Besides, this patient was tested negative for relevant lab tests or was not tested.”

The meaning of this sentence is unclear. What kind of person does 'this patient' refer to? If this patient are the people have clinical symptoms, the meaning of sentence is vague.

**Respond:** We added the diagnose criteria and revised the sentence.

2. **Response to Results**

2.1 This is a good research of the HFRS case from 1963 to 2020 showing epidemiology according to various patterns through spatio-temporary analysis. Epidemiological trends, geographic distributions, and dynamic change of seasonal distributions are well analyzed within a Province. Notation of numbers or English needs to be improved.

**Respond:** We invited an English native speaker to revise the whole manuscript.

**2.2** - In Figure 3, the name of joint region (Shengzhou county, Xinchang county, and Tiantai County) must be written in figure. It is difficult for readers to intuitively identify the area.

**Respond:** We added the name of joint region in figure 3.

3. **Response to Conclusions**

3.1 The conclusion is mostly make sense and acceptable, but the connection with climate seems to be overinterpreted. The year-round surrounding rainfall and humility and association with HFRS will require further analysis of the direct correlation.

**Respond:** The relationship between disease and climate is a flaw in this study. In future studies, more research on influencing factors and the correlation of diseases will be included. We added “the year-round surrounding rainfall and humility and association with HFRS will require further analysis” in the discussion section.

**3.2** - Virus name must be corrected. (HTNV)

- The reference style shall be unified.

- Line number must also be filled in.

**Response**: We had changed the HTNV in the manuscript, revised the reference style, and added line number.

Thank you again for your valuable advices.

**Response to Reviewer #2:**

**1.** The paper is mostly informative. It is well written. It isn’t to long, so the readers can read it.
I suggest to shortness the abstract section. It’s too long. There are enough or adequate tables and figures.
**Respond**: We shortened the abstract section.

2. Instead of the world “sex” is better to use “gender”

**Respond**: We had corrected the world “sex” to “gender”.

3.In the discussion section the authors try to explain the gender differences. In this paragraph I suggest to add this reference: In the discussion section the authors try to explain the gender differences. In this paragraph I suggest to add this reference:

Puca E, Pipero P, Harxhi A, Abazaj E, Gega A, Puca E, Akshija I (2018) The role of gender in the prevalence of human leptospirosis in Albania. J Infect Dev Ctries 12:150-155. doi: 10.3855/jidc.9805

The authors are correct with citation of the study limitations.

I suggest to add and this reference in their paper

Puca E, Qato M, Pipero P, Akshija I, Kote M, Kraja D. Two cases of imported hemorrhagic fever with renal syndrome and systematic review of literature. Travel Med Infect Dis. 2019 Mar-Apr; 28:86-90. doi: 10.1016/j.tmaid.2018.07.010. Epub 2018 Aug 13. PMID: 30114480.

**Respond**: We added this references in the discussion section.

Thank you again for your valuable advices.

**Response to Reviewer #3:**

**1.Response to Methods**:

1.1 methods employed are state-of the art;
-Page 1: please, change the name Dobrava to Dobrava-Belgrade virus

**Respond**: We had revised it.

1.2 Page 2: please, include the reference for the sentence: “In the recent ten years, annual HFRS incidence was below 1 per 100,000 population.”

**Response**: We added the relative reference.

1.3 Page 2: in the sentence “Data from 1963 to 1990 and data from 1991 to 2004 were obtained from Zhejiang CDC archives.” There is no need to split the time period, merge to “from 1963 to 2004”; the explanation is in the following sentence and this one is just confusing
- Please, when referring to campaigns and policy changes, include brief explanation in addition title, e.g  what is “Two Mountains Theory” (page 3)?

**Respond**: We revised relative sentences.

2. **Response to Results**: the results could be presented in a more clear way:

**Respond**: We rewrote some sentences of the result section.

**3. Response to Conclusions**

Please, present results in a more clear way; figure captions need to be more self-explanatory, especially Figures 3, 5, 6.

**Respond**: We revised the figure caption of Figure 3, 5, 6.

4. Please, consider presenting joinpoint analysis results in the main text, albeit in a more structured way than currently, whereas Tables1, 3, 4 could be moved to the supplementary material.

Editorial and Data Presentation Modifications?

**Respond**: We presented jointpoint analysis results in the main text and moved table 1, 3, 4 to the supplementary material.

5. **Response to Summary and General Comments**

The manuscript presents original research on a significant topic – by authors’ account it is the largest-scale epidemiology study on hemorrhagic fever with renal syndrome reported so far. I believe it is of interest for the readership of the PLOS Neglected Tropical Diseases. The manuscript is nicely written, statistical methods employed are state-of the art, however, the results could be presented in a more clear way, it is a bit difficult to follow. I suggest the manuscript to be accepted for publication, with some minor changes and suggestions for improvement.

**Response:** We rewrote some sentences of the result section.

Thank you again for valuable comments.

We have tried our best to improve the manuscript and made some changes in the manuscript. We appreciate for Editors/Reviewers’ warm work earnestly, and hope that the correction will meet with approval.
Once again, thank you very much for your comments and suggestions.

Yours sincerely,
Jimin Sun
